# Combined immune checkpoint inhibitor therapy with nivolumab and ipilimumab causing acute-onset type 1 diabetes mellitus following a single administration: two case reports

**DOI:** 10.1186/s12902-019-0467-z

**Published:** 2019-12-23

**Authors:** Marco Zezza, Christophe Kosinski, Carine Mekoguem, Laura Marino, Haithem Chtioui, Nelly Pitteloud, Faiza Lamine

**Affiliations:** 1 Service of Endocrinology, Diabetes and Metabolism, Lausanne University Hospital (CHUV), University of Lausanne, Av. de La Sallaz 8, 1011 Lausanne, Switzerland; 2 Service of Clinical Pharmacology, Lausanne University Hospital (CHUV), University of Lausanne, Lausanne, Switzerland

**Keywords:** Immune checkpoint inhibitor, Autoimmune adverse events, Endocrinopathies, Type 1 diabetes

## Abstract

**Background:**

The use of immune checkpoint inhibitor (ICI) therapy is becoming a standard of care for several cancers. Monoclonal antibodies targeting cytotoxic T-lymphocyte antigen-4 (CTLA-4) and programmed cell death protein 1 (PD-1) or its ligand (PD-L1) cause a broad spectrum of autoimmune adverse events. ICI-induced type 1 diabetes mellitus (T1DM) is extremely rare (< 1%) but potentially life-threatening. It appears to be more common with PD-1 blockade (or combination immunotherapy) than with anti-CTLA-4 therapy, often during the first three to six months of therapy.

**Cases presentation:**

We report an acute onset T1DM with severe inaugural diabetic ketoacidosis (DKA) and remarkably elevated Glutamic Acid Decarboxylase antibody (GADA) titres following a single administration of combined ICI therapy with nivolumab (anti-PD-1) and ipilimumab (anti-CTLA-4) in two adult patients with advanced metastatic melanoma. In these cases, the time to diabetes onset was remarkably short (two and five weeks), and one presented with fulminous T1DM in a previous long-standing type 2 diabetes mellitus.

**Conclusions:**

Oncological patients treated with combination therapy of anti-PD-1 and anti-CTLA-4 can develop a particular pattern of T1DM, with very rapid onset within a few weeks after starting ICI therapy, even in the presence of an existing type 2 diabetes. ICI-induced T1DM is a medical emergency in presence of severe inaugural DKA and requires a collaboration between specialists and primary care physicians, as well as patient education, for early diagnosis and supportive care.

## Background

Immune checkpoint inhibitors (ICI) are the major breakthrough in cancer therapy in the last decade. Administration of monoclonal antibodies targeting cytotoxic T-lymphocyte antigen-4 (CTLA-4), programmed cell death protein 1 (PD-1) or its ligand (PD-L1) enhances the immune system response against tumour cells. ICI improve survival in a subset of cancer patients [1–4]. Nevertheless, ICI are frequently associated with immune-related adverse events (irAE) [5], and the combination of anti-CTLA-4 and anti-PD-1 or PD-L1 rises both the intensity and frequency rates of irAE up to 60% corresponding to a two or three-fold increase compared to single-agent ICI therapy [6]. ICI-induced irAE affect all organs, but gastrointestinal tract, liver, skin and endocrine systems are more often affected [5]. The most common ICI-associated endocrinopathies are thyroid disorders and hypophysitis [7]. ICI-induced type 1 diabetes mellitus (T1DM) is uncommon, with a reported frequency ranging from 0.2% in randomised clinical studies [7] to 0.9% in real-life setting [8].

We describe herein two patients with metastatic melanoma presenting with severe diabetic ketoacidosis (DKA) and positive β-cell autoantibodies shortly after a single infusion of ICI combination therapy with ipilimumab (IPI) and nivolumab (NIVO). Interestingly in the first case, we observed an acute onset of T1DM in a long-standing type 2 diabetes mellitus (T2DM) concurrently to immune-checkpoint inhibitor-induced pancreatic injury.

## Cases presentation

### Case 1

A 60-year-old man with a 10 years history of T2DM was diagnosed with a metastatic cutaneous melanoma and was started on ICI treatment, with IPI (3 mg/kg every 3 weeks for four cycles) and NIVO (1 mg/kg every 3 weeks for four cycles). He had an optimal glycaemic control (HbA1C 6.2% two months before ICI initiation) under oral antidiabetic medication (metformin, sitagliptin and gliclazide) **(**Table 1**)**. Two weeks after the first ICI infusion, he was admitted to the hospital with vomiting, polyuria, polydipsia, hyperglycaemia (46 mmol/L), severe acidosis (pH 6.9) and presence of ketones bodies in urine (> 7.8 mmol/L) (Table 1). Treatment protocol for DKA with continuous intravenous insulin was initiated with a favourable outcome within 8 hours. Further workup revealed an elevated Glutamic Acid Decarboxylase antibody (GADA) titre 19,770 IU/ml (*N* < 10) in line with an autoimmune T1DM. The pituitary function was in the normal range. Concurrent plasma lipase level was substantially increased (988 UI/l). According to the ongoing protocol used by our institution’s oncologists which was based on Common Terminology Criteria for Adverse Events (CTCAE) of the National Institute of Health, immune-checkpoint inhibitor-induced pancreatic injury was suspected although abdominal CT-scan was unremarkable. The oncologists decided to discontinue IPI and NIVO after this first dose and to start with prednisone 1 mg/kg/day with a tapered dosage schedule over 14 weeks. Lipase levels decreased by 60% within 48 h, and maintained close to the upper range within 14 days. The patient was discharged on multiple daily insulin injections, with a dosage of 0.6 U/Kg/day. Eighteen months later, cancer disease was in complete remission, and diabetes remained insulin-dependent (0.4 U/kg/day) and relatively well controlled (HbA1C 7.6%).

**Table 1:** Clinical and biochemical data at diabetes onset in our index cases and cases reported in the literature

	Case n° 1	Case n° 2	Changizzadeh et al. [9]	Gunawan et al. [10]	Lowe et al. [11]
Age (years) / Gender	60/M	80/F	42/M	52/M	54/M
History of diabetes	T2DM	No	No	No	No
Familial history of diabetes	No	No	No	NR	NR
Neoplasia	Melanoma stage IV	Melanoma stage IV	Melanoma stage IV	Melanoma stage IV	Melanoma stage IV
Prior corticosteroid administration	No	No	No	Yes^a^	Yes^b^
ICI regimen	IPI (3 mg/kg) and NIVO (1 mg/kg) every 3 weeks	IPI (3 mg/kg) and NIVO (1 mg/kg) every 3 weeks	IPI (3 mg/kg) and NIVO (1 mg/kg) every 3 weeks	IPI (3 mg/kg) and NIVO (1 mg/kg) every 3 weeks	IPI (3 mg/kg) and NIVO (1 mg/kg) every 3 weeks
Time to T1DM onset	2 weeks (1 cycle)	5 weeks (1 cycle)	12 weeks (3 cycles)	11 weeks (3 cycles)	19 weeks (3 cycles)
DKA	Yes	Yes	Yes	No	Yes
Plasma glucose (mmol/L)	46	48.4	40.4	20.8	NR
Ketones bodies (mmol/L)					
Plasma	NA	NA	NR	2.4	NR
Urine	> 7.8	> 7.8	NR	NR	0.40
HbA1C (%)	7.5	NA	6.5	7.7	NR
C-Peptide (nmol/L)	NA	NA	NR	0.05 (N: 0.4–1.5)	NR
T1DM-related autoantibodies	+ (GADA, ICA, IA2)	+ (GADA, ICA) IA2 -	- (GADA, IA2, ZnT8A)	- (GADA, IA2, ZnT8A)	(GADA)
Other immune-related endocrine toxicities	None	Autoimmune thyroiditis	None	Hypophysitis, Diabetes insipidus	Autoimmune thyroiditis Hypophysitis
Non endocrine immune related toxicities	Presumed immune-checkpoint inhibitor-induced pancreatic injury	None	Colitis	None	Hepatitis, Colitis
Definitive discontinuation of ICI	Yes	Yes	Yes	Yes	Yes

^a^Indication: autoimmune hypophysitis; Dexamethasone 8 mg twice a day for 24 h and then hydrocortisone 30 mg/day were started five weeks before T1DM onset. ^b^Indication: autoimmune hepatitis and colitis; Prednisone was started ten weeks before T1DM and discontinued before T1DM onset. Abbreviations: *M* Male, *F* Female, *ICI* Immune checkpoint inhibitors, *IPI* Ipilimumab, *NIVO* Nivolumab, *W* Week; *T2DM* Type 2 diabetes mellitus, *T1DM* Type 1 diabetes mellitus, *NA* Not analysed, *NR* Not reported

### Case 2

An 80-year-old woman without a history of diabetes was started on IPI-NIVO for advanced metastatic melanoma. Casual plasma glucose performed 3 months before ICI treatment was 8.3 mmol/L. Three weeks after the first infusion, she presented with transient autoimmune thyroiditis **(**Table 1**)**. Immunotherapy was suspended and thyroid function recovered within 2 weeks. Five weeks after this first ICI administration, she was admitted to the emergency unit for acute mental confusion developed 24 hours before admission. An infectious aetiology was excluded. In the setting of severe hyperglycaemia (48.4 mmol/L), severe acidosis (pH 7.1) and urinary ketones bodies (> 7.8 mmol/L), adequate treatment was started leading to DKA resolution within 6 hours. GADA titre was high (2000 IU/ml, *N* < 10) consistent with autoimmune T1DM (Table 1). Multiple daily insulin injections were started. Cerebral CT-scan performed 24 hours later showed a progression of cerebral metastases and a subarachnoid haemorrhage. The patient died 3 weeks later due to disease progression.

## Discussion

The most striking finding in our two cases is the remarkably short time to onset of autoimmune T1DM with severe DKA following a single infusion of a combination ICI therapy with anti-CTLA-4 and anti-PD-1. ICI-induced T1DM is specific to anti-PD-1/PD-L1 use [9–27] as PD-L1 is expressed in β-cells, and PD-1 receptor is expressed by T cells. The interaction PD-1/PD-L1 inhibits the activation of autoreactive T-cells, thereby protecting against autoimmune diabetes [28]. Non-obese diabetic (NOD) mice deficient in PD-1 rapidly develop autoimmune diabetes [29]. Injection of anti-PD-1 or anti-PD-L1 in NOD mice caused the development of diabetes with extensive destructive insulitis mediated by specific CD8 T cells [30]. In clinical settings, the occurrence of T1DM under ICI combination therapy has been rarely reported [9–11, 31]. It is characterised by an earlier onset compared to T1DM induced by single-agent anti-PD-1/PD-L1 therapy. Rapid onset T1DM occurring within two to 5 weeks after a single infusion of ICI combination, as observed in our cases is very peculiar. To our knowledge, the largest reported case series of ICI-induced T1DM included twenty-seven patients: twenty-two of them were on anti-PD-1 or anti-PD-L1 alone, and only five were on IPI-NIVO combination. Among these five patients, only one presented T1DM following a single exposition to IPI-NIVO and was diagnosed within 5 weeks. In the aforementioned case series, the median time to onset was 20 weeks, and most cases associated with single-agent anti-PD-1/PD-L1 therapy occurred after 10 weeks [8]. The unexpected acute onset of autoimmune T1DM observed in our cases, especially the first one, could be explained by an extensive acute insulitis as reflected by the unusual remarkably elevated GADA titres, which were ten to forty-fold higher than those reported in the literature [32]. GADA, which is a critical marker of autoimmune T1DM, is usually detected in 30 to 50% of ICI-induced diabetes cases [8, 12, 33]. Correlation between GADA positivity and a shorter time to onset of diabetes was demonstrated in a recent study involving twenty-four patients treated with anti-PD-1 [34]. The median time to onset under anti-PD-1 treatment was 3 weeks in GADA-positive individuals vs 12.5 weeks in GADA negative individuals. Moreover, higher GADA titres may be linked to the earlier onset and greater clinical severity of diabetes in patients who presented a fulminant T1DM occurring either spontaneously or under anti-PD-1 treatment [35]. Presence of β-cell autoantibodies before ICI initiation could be a risk factor of diabetes occurrence. However, this parameter was poorly assessed in the literature and was not tested in our patients since it is not recommended in routine clinical practice [36, 37]. It is noteworthy that nearly 50% of anti-PD-1/PD-L1-induced inaugural DKA cases are GADA negative, but they also require long-term insulin therapy like the classic autoimmune T1DM [38, 39]. GADA-negative T1DM under anti-PD-1 therapy could be triggered by a sudden and major activation of beta-cell reactive CD8+ T-cell clones without the involvement of humoral immunity in the short time frame before overt diabetes [39].

The presence of pre-existing T2DM such as in case 1 does not preclude the onset of ICI-induced T1DM. Autoimmune diabetes superimposed on T2DM in the setting of ICI is extremely rare with only two cases being reported so far in the setting of ICI combination therapy with relatively early onset (three and 4 weeks after ICI initiation respectively) [32].

Regarding associated factors, other irAE can occur in up to 70% of patients before or concurrently with ICI-induced diabetes. Thyroiditis with transient thyrotoxicosis is the most frequent irAE with a prevalence rate ranging from 23 to 40% in ICI-induced T1DM patients [8, 12, 14, 17]. Immune-checkpoint inhibitor-induced pancreatic injury was reported in nearly 30% of patients on the day of ICI-induced diabetes diagnosis [8]. Genetic susceptibility for ICI-induced diabetes including potential predisposing HLA genotypes is still a matter of debate [8, 11, 14, 17–20, 22, 24]. Accordingly, HLA types were not routinely assessed in our cases.

### Clinical practice issues

ICI-induced T1DM is a medical emergency since severe inaugural DKA occurs in 60 to 85% of cases [12]. This condition can be particularly life-threatening in these frail cancer patients who are older than the majority of classic T1DM patients [8]. As for classic T1DM, ICI-induced diabetes is also caused by a severe insulin deficiency with low or undetectable levels of C-peptide [12] and require long-term insulin therapy. The diagnosis can be initially challenging because of the very rapid onset of hyperglycaemia even after the first ICI administration. Typical symptoms such as polyuria and polydipsia can thus be missing as in our second case, and HbA1C can be relatively low as observed in our first case [9–11]. Moreover, patients may present with non-specific complaints that can be linked to either cancer complications as observed in our second case or gastrointestinal toxicity, which occurs more frequently than diabetes [8–10].

According to current guidelines, fasting venous blood glucose should be tested only in patients receiving anti-PD-1/PD-L1 therapy, with close monitoring especially during the first 6 months, which is the highest risk period for endocrine toxicity onset [37]. In our opinion, patient education for rapid recognition of DKA symptoms and urgent medical referral is more useful than serial monitoring of glucose levels since ICI-induced DKA can occur within a very short time frame. For patients with pre-existing diabetes and treated with anti-PD-1/PD-L1, self-monitoring of blood glucose should be proposed or reinforced [37]. If ICI-induced T1DM occurs, insulin therapy should be immediately started according to standards of care. Assessment of HbA1C and antibody panel (GADA, anti-IA2 and anti-ZnT8 if available), as well as serum insulin and C-peptide whenever it is possible, is useful for phenotyping this rare entity, but not essential. It is also important to evaluate the pituitary function not to overlook a concurrent hypophysitis or adrenalitis that may partially mimic DKA symptoms [11]. ICI should be temporarily stopped until DKA resolution and then be rapidly resumed [26, 37]. Unlike the majority of severe non-endocrine ICI toxicities that are responsive to corticosteroid therapy, ICI-induced T1DM is not reversed by corticosteroids [9–11, 13, 24, 27, 37] and requires long-term insulin therapy. As ICI are used in early oncological disease nowadays, the overall survival could be improved, and thus glycaemic target should be individualised. In patients with limited overall survival due to advanced oncological situation, glycaemic targets should be less stringent, with HbA1C < 8.0% [37], but in other cases, the “standard” HbA1C-goal should be targeted [40].

To conclude, oncological patients treated with combination therapy of anti-PD-1/PD-L1 and anti-CTLA-4 can develop a particular pattern of T1DM, with very rapid onset within a few weeks after starting ICI therapy, even with a pre-existing T2DM. Reporting to pharmacovigilance system is highly recommended to gather clinical information and help to identify better the natural history of this rare but potentially life-threatening adverse event. Assessment of T1DM antibodies at baseline could be useful to offer personalised monitoring for patients at high risk of ICI-induced diabetes. Further studies are warranted to determine predictive markers of ICI-induced T1DM.

## Abbreviations

CTLA-4: Cytotoxic T-lymphocyte antigen-4
DKA: Diabetic ketoacidosis
GADA: Glutamic acid decarboxylase antibody
ICI: Immune checkpoint inhibitor
IPI: Ipilimumab
irAE: Immune-related adverse events
NIVO: Nivolumab
NOD: Non-obese diabetic
PD-1: Programmed cell death protein 1
PD-L1: Programmed cell death protein 1 ligand
T1DM: Type 1 diabetes mellitus
T2DM: Type 2 diabetes mellitus
